# Supplementary material for: Injectable hydrogel bioelectrostimulator for wireless deep brain neuromodulation
Source: Nat Commun. 2026 Feb 4;17:4526. doi: 10.1038/s41467-026-69226-1 (PMC13194742; doi:10.1038/s41467-026-69226-1)
Supplement: Supplementary file 1 — Supplementary Information [file 41467_2026_69226_MOESM1_ESM.pdf]

Injectable hydrogel bioelectrostimulator for wireless deep brain neuromodulation

Ming Yang[1#], Wenliang Liu[1#], Ping Chen[1#], Zhuang Liu[2#], Renyuan Sun[1#], Baochun Xu[3], Qiong Wang[4], Bingqing Xue[5], Chuan Gao[1], Jiahui She[1], Chong Ma[1], Dingke Zhang[1], Zhikun Li[1], Nanxi Yi[1], Donghui Zhang[5], Jiexiong Feng[4], Cunjiang Yu[3,6,7,8*], Jie Wang[2,9*], and Zhiqiang Luo[1,4,10*]

[1] National Engineering Research Center for Nanomedicine, College of Life Science and Technology, Huazhong University of Science and Technology, Wuhan, 430074, China.

[2] Department of Neurology, Songjiang Hospital Affiliated to Shanghai Jiao Tong University School of Medicine, Shanghai, 201600, China.

[3] Department of Electrical and Computer Engineering, University of Illinois, Urbana-Champaign, Urbana, IL 61801, USA.

[4] Department of Pediatric Surgery, Tongji Hospital, Tongji Medical College, Huazhong University of Science and Technology, Wuhan, 430030, China.

[5] Stem cells and Tissue Engineering Manufacture Center, School of Life Science, Hubei University, Wuhan, 430062, China.

[6] Department of Materials Science and Engineering, University of Illinois, Urbana-Champaign, Urbana, IL 61801, USA.

[7] Department of Mechanical Science and Engineering, University of Illinois, Urbana-Champaign, Urbana, IL 61801, USA

[8] Department of Bioengineering, Materials Research Laboratory, Beckman Institute for Advanced Science and Technology, Nick Holonyak Micro and Nanotechnology Laboratory, University of Illinois, Urbana-Champaign, Urbana, IL 61801, USA

[9] Shanghai Key Laboratory of Emotions and Affective Disorders, Songjiang Research Institute, Songjiang Hospital Affiliated to Shanghai Jiao Tong University School of Medicine, Shanghai, 200062, China.

[10] Research Center for Intelligent Fiber Devices and Equipment, State Key Laboratory of New Textile Materials and Advanced Processing, Huazhong University of Science and Technology, Wuhan, 430074, China.

[#] These authors contributed equally: Ming Yang, Wenliang Liu, Ping Chen, Zhuang Liu, Renyuan Sun.

***Corresponding Authors**

cunjiang@illinois.edu (C. Yu)

jie.wang@shsmu.edu.cn (J. Wang)

zhiqiangluo@hust.edu.cn (Z. Luo)

Content

Supplementary Fig. 1. Pyrrole polymerization by GOx-HRP cascade.

Supplementary Fig. 2. UV-Vis spectra of polypyrrole polymerized in the presence of glucose.

Supplementary Fig. 3. Zeta potential characterization of PEDOT:PSS and PPy dispersions.

Supplementary Fig. 4. Optical microscopy image of ICHs.

Supplementary Fig. 5. FTIR and Raman spectra of ICHs.

Supplementary Fig. 6. Rheological properties of the ICHs.

Supplementary Fig. 7. Glucose-dependent gelation and mechanical properties of ICHs.

Supplementary Fig. 8. Electrical conductivity of ICHs.

Supplementary Fig. 9. Frequency-dependent impedance characterization of ICHs.

Supplementary Fig. 10. Charge storage performance of ICHs.

Supplementary Fig. 11. Charge injection performance of ICHs.

Supplementary Fig. 12. Finite element model of the scalp-skull-brain system for WES simulation.

Supplementary Fig. 13. Finite element simulation of interfacial polarization and field localization at the ICH-tissue interface.

Supplementary Fig. 14. Frequency-dependent specific absorption rate (SAR).

Supplementary Fig. 15. Frequency-dependent voltage and current output of the capacitive coupling system and ICH.

Supplementary Fig. 16. Current output of capacitive coupling system and ICH with varying input voltage.

Supplementary Fig. 17. Fabrication of the cell culture chamber with patterned indium tin oxide (ITO) electrodes.

Supplementary Fig. 18. Live-dead staining of PC12 cells cultured on ICH under WES with different current intensities.

Supplementary Fig. 19. Viability of PC12 cells cultured on ICH with and without WES.

Supplementary Fig. 20. Flow cytometry analysis of PC12 cell viability under ICH (-) conditions.

Supplementary Fig. 21. Thermal effects of WES.

Supplementary Fig. 22. Power spectral density (PSD) of GPi neuronal activity.

Supplementary Fig. 23. Cellular Fos (c-Fos) expression in the cortical region of rats.

Supplementary Fig. 24. Multi-channel neural recordings from primary motor cortex (M1).

Supplementary Fig. 25. Time-frequency spectrogram of M1 neuronal activity.

Supplementary Fig. 26. Joint inter-spike interval (ISI) distribution of M1 neuronal firing patterns.

Supplementary Fig. 27. Representative un-cropped confocal images of Iba-1 and GFAP immunostaining used for quantification.

Supplementary Fig. 28. Open field test of PD rats during 4-week treatment.

Supplementary Fig. 29. ICH-mediated DBS enhances neuronal survival.

Supplementary Fig. 30. Immunofluorescence analysis of Iba-1^{+} microglia.

Supplementary Fig. 31. Immunofluorescence analysis of GFAP^{+} astrocytes.

Supplementary Fig. 32. ICH-mediated DBS enhances BDNF expression.

Supplementary Fig. 33. Structural MRI after PD modeling and injection of ICH.

Supplementary Fig. 34. Whole-brain functional connectivity analysis.

Supplementary Fig. 35. Voxel-based morphometry (VBM) analysis of gray and white matter after ICH-mediated DBS.

Supplementary Table 1. Brain region index and anatomical labels.

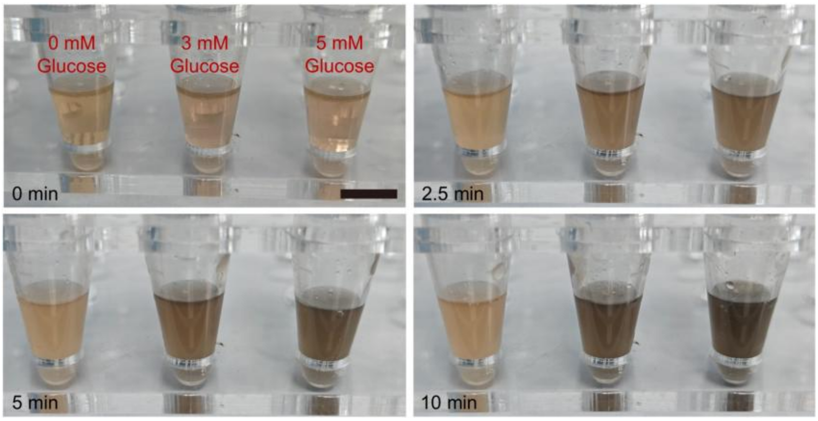

Supplementary Fig. 1. Pyrrole polymerization by GOx-HRP cascade. The solution gradually darkens with increasing glucose concentration and reaction time, indicating the progression of pyrrole polymerization. Scale bar, 1 cm. GOx, glucose oxidase; HRP, horseradish peroxidase.

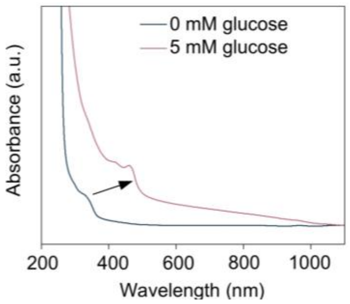

Supplementary Fig. 2. UV-vis-NIR spectra of polypyrrole polymerized in the presence of glucose. The absorption peak shifts from 300-350 nm to 400-450 nm with increasing glucose concentration, indicating an extension of the conjugation length or alterations in the electronic structure of polypyrrole (PPy) during polymerization.

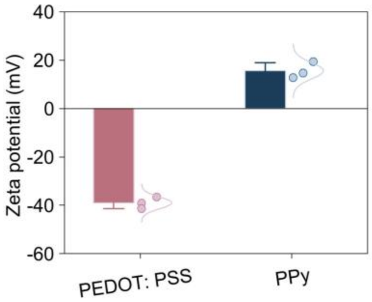

Supplementary Fig. 3. Zeta potential characterization of PEDOT:PSS and PPy dispersions. Zeta potential measurements confirming opposite surface charges of PPy and PEDOT:PSS under neutral aqueous conditions (n = 3 independent experiments). PPy exhibited a positive zeta potential of approximately +18 mV, while PEDOT:PSS showed a negative zeta potential of approximately -40 mV. Data are presented as the mean ± standard deviation.

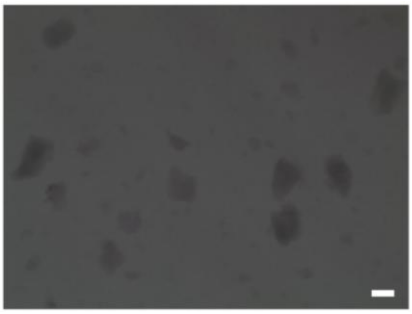

Supplementary Fig. 4. Optical microscopy image of ICHs. The image shows dispersed hydrogel microgels formed through enzymatic polymerization. Scale bar, 100 µm.

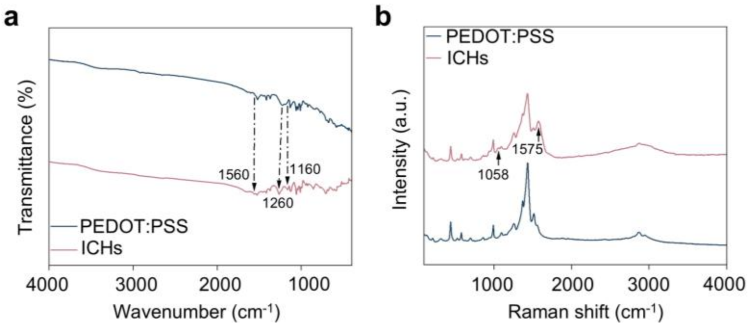

Supplementary Fig. 5. FTIR and Raman spectra of ICHs. a, FTIR spectra revealing structural changes in PEDOT:PSS due to pyrrole polymerization. **b,** Raman spectra showing PPy formation with characteristic C=C/C=N and C-H/C-N bands.

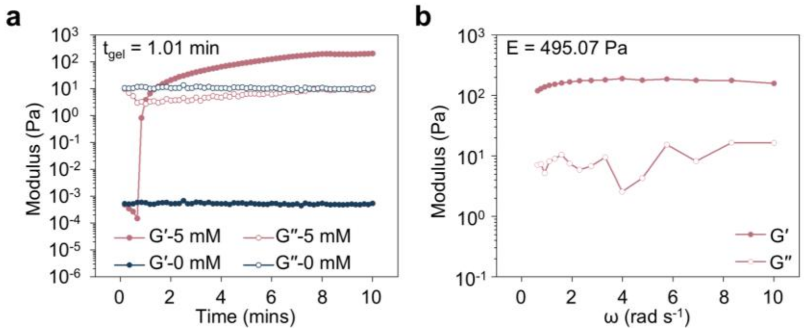

Supplementary Fig. 6. Rheological properties of the ICHs. a, Time sweep of the storage modulus (G′) and loss modulus (G″) of ICHs, indicating gelation with a time of 1.01 min. **b**, Frequency sweep of the ICHs after gelation, showing Young's modulus of 495.07 Pa.

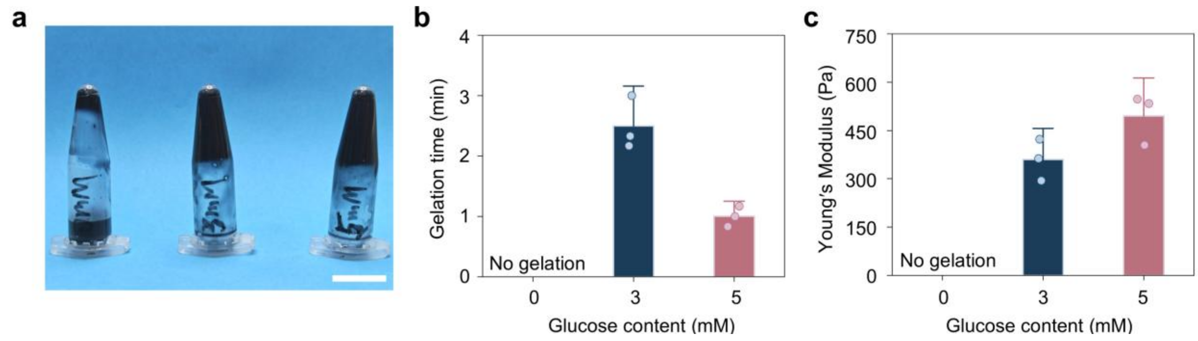

Supplementary Fig. 7. Glucose-dependent gelation and mechanical properties of ICHs. a, Photographs of ICHs precursors after incubation in the presence of different glucose concentrations (0, 3, and 5 mM), showing gelation at physiological glucose levels. Scale bar, 1 cm. **b**, Gelation times of ICHs precursor at different glucose concentrations (n = 3 independent experiments). **c**, Young's modulus of ICHs at different glucose contents (n = 3 independent experiments). Data are presented as the mean ± standard deviation in (**b**, **c**).

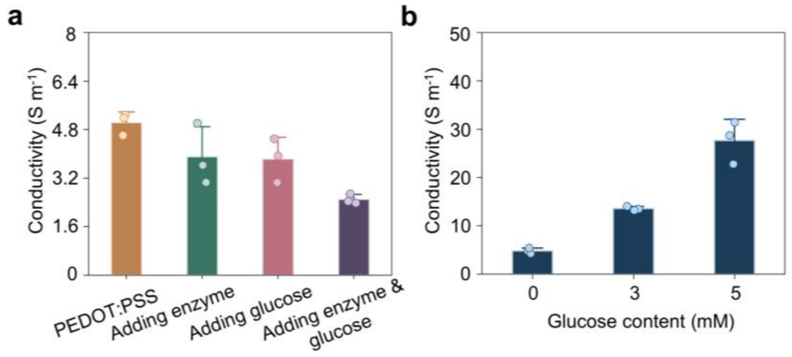

Supplementary Fig. 8. Electrical conductivity of ICHs. a, Conductivity of non-polymerized precursor mixtures without pyrrole monomers (n = 3 independent experiments), including PEDOT:PSS alone, PEDOT:PSS with GOx/HRP only, PEDOT:PSS with glucose only, and PEDOT:PSS with both GOx/HRP and glucose, all showing low conductivity (< 6 S m^{-1}). **b**, Conductivity of the ICHs increased with higher glucose concentrations, reaching ~30 S m^{-1} at 5 mM glucose (n = 3 independent experiments). Data are presented as the mean ± standard deviation.

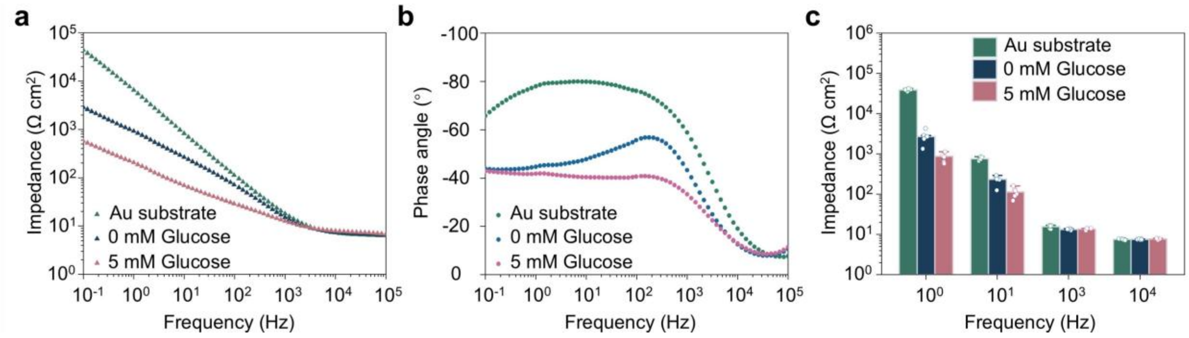

Supplementary Fig. 9. Frequency-dependent impedance characterization of ICHs. a, Bode plot of impedance, showing a reduction in impedance across the entire frequency rang following glucose-induced PPy formation. **b**, Phase angle spectra indicating reduced capacitive behavior at low frequencies in ICHs formed under 5 mM glucose, consistent with decreased capacitive reactance. **c**, Impedance values at selected frequencies, further confirming enhanced charge transport with increasing glucose concentration, as compared to gold (Au) electrodes (n = 3 independent experiments). Data are presented as the mean ± standard deviation in (**c**).

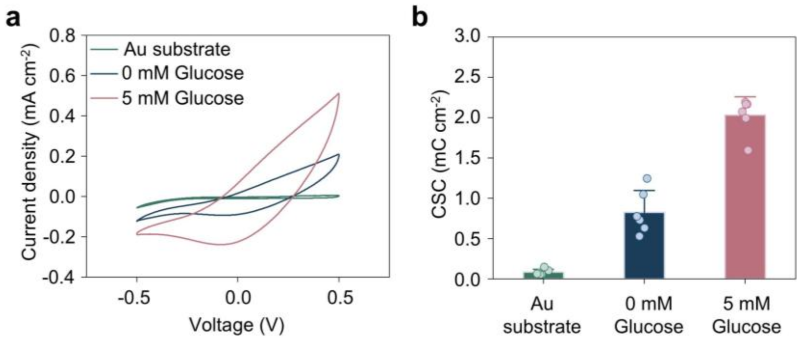

Supplementary Fig. 10. Charge storage performance of ICHs. a, Cyclic voltammetry curves show enhanced current response in ICHs with glucose-induced PPy formation. **b**, Charge storage capacity (CSC) comparison demonstrates enhanced charge storage upon glucose-triggered PPy formation (n = 6 independent experiments). Data are presented as the mean ± standard deviation in (**b**).

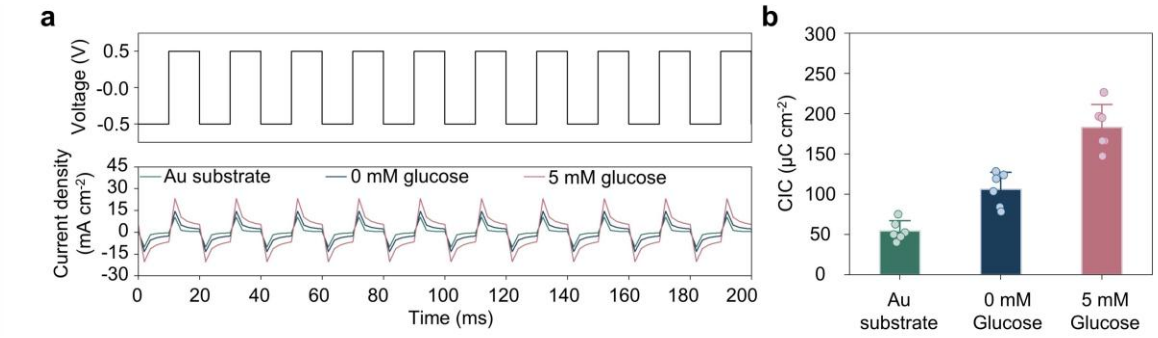

Supplementary Fig. 11. Charge injection performance of ICHs. a, Current density responses of ICHs and Au electrodes under symmetric biphasic voltage pulses (±0.5 V), showing enhanced charge delivery in the 5 mM glucose group. **b**, Charge injection capacity (CIC) values derived from the pulse responses, indicating improved charge injection efficiency following glucose-induced PPy formation (n = 6 independent experiments). Data are presented as the mean ± standard deviation in (**b**).

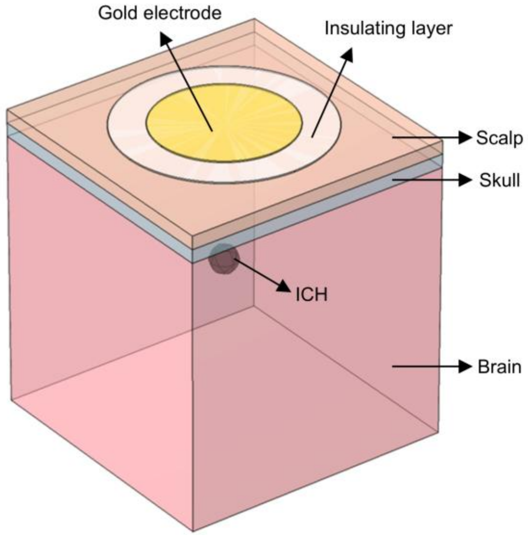

Supplementary Fig. 12. Finite element model of the scalp-skull-brain system for WES. Schematic of the 3D model constructed for simulating electric-field and thermal distributions during WES. The model consists of a multilayer structure representing scalp, skull, and brain tissues, with an insulated metal transmitter and an implanted ICH located in the brain tissue.

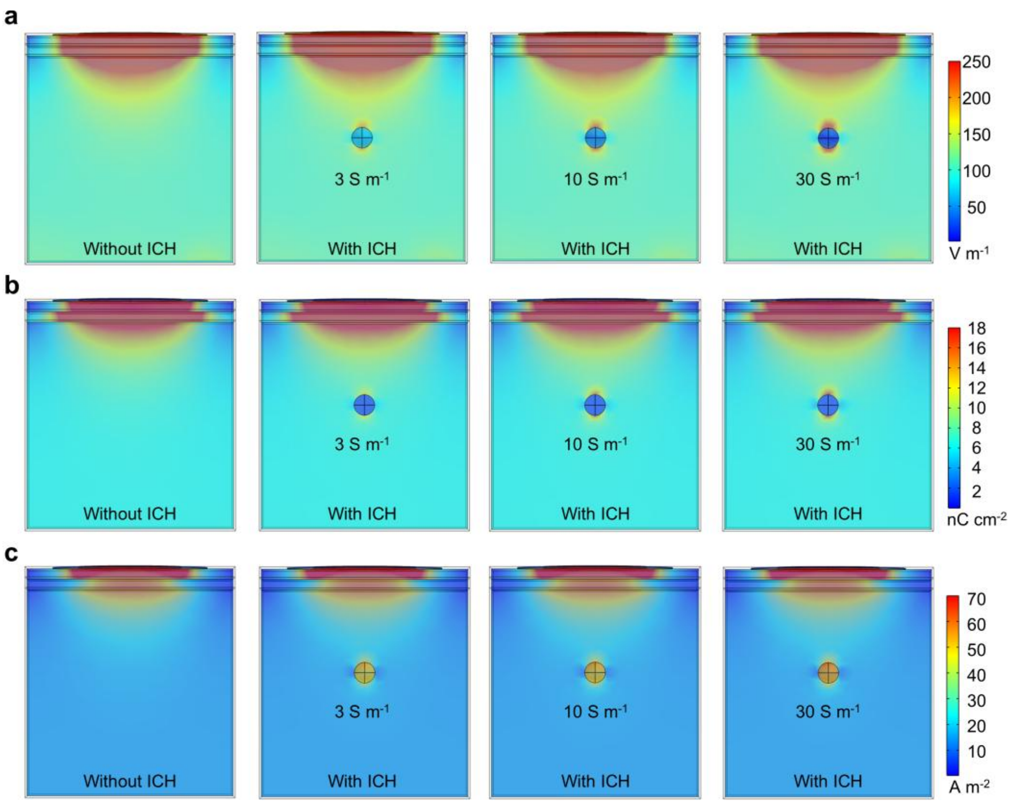

Supplementary Fig. 13. Finite element simulation of interfacial polarization and field localization at the ICH-tissue interface. a, Simulated electric field intensity distribution in the multilayer head model without and with ICHs of different conductivities (3, 10, and 30 S m^{-1}). The introduction of ICHs leads to localized field enhancement around the hydrogel-tissue interface. **b**, Corresponding surface charge density maps reveal charge accumulation at the ICH boundary, which increases with hydrogel conductivity. **c**, Local current density distribution showing enhanced conduction pathways near the implanted ICH region, consistent with interfacial polarization-induced field focusing effects.

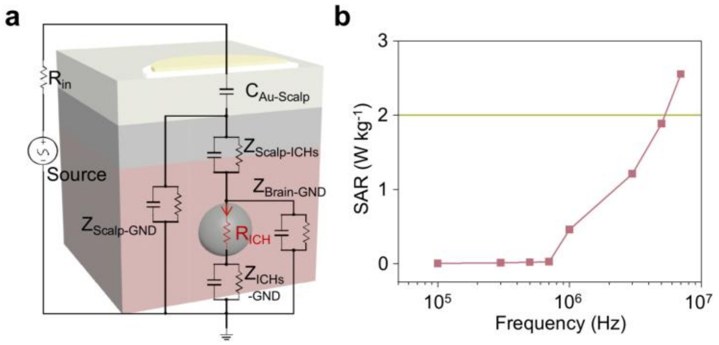

Supplementary Fig. 14. Frequency-dependent specific absorption rate (SAR). a, Frequency-adaptive circuit model integrating capacitive coupling, multi-tissue impedance, and brain admittance with interfacial polarization mechanisms. **b**, SAR values as a function of frequency, indicating SAR remains below the biosafety threshold at frequency of 5 MHz.

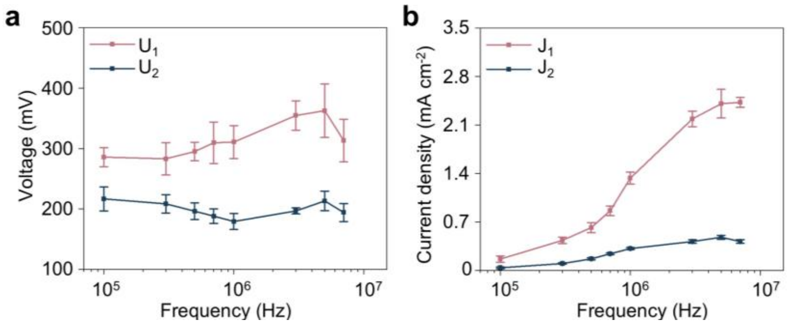

Supplementary Fig. 15. Frequency-dependent voltage and current output of the capacitive coupling system and ICH. a, Measured output voltages of the entire system and ICHs across frequencies under a 2.5 V input (n = 3 independent experiments). **b**, Measured output current densities of the complete system and ICHs across frequencies under a 2.5 V input (n = 3 independent experiments). Data are presented as the mean ± standard deviation.

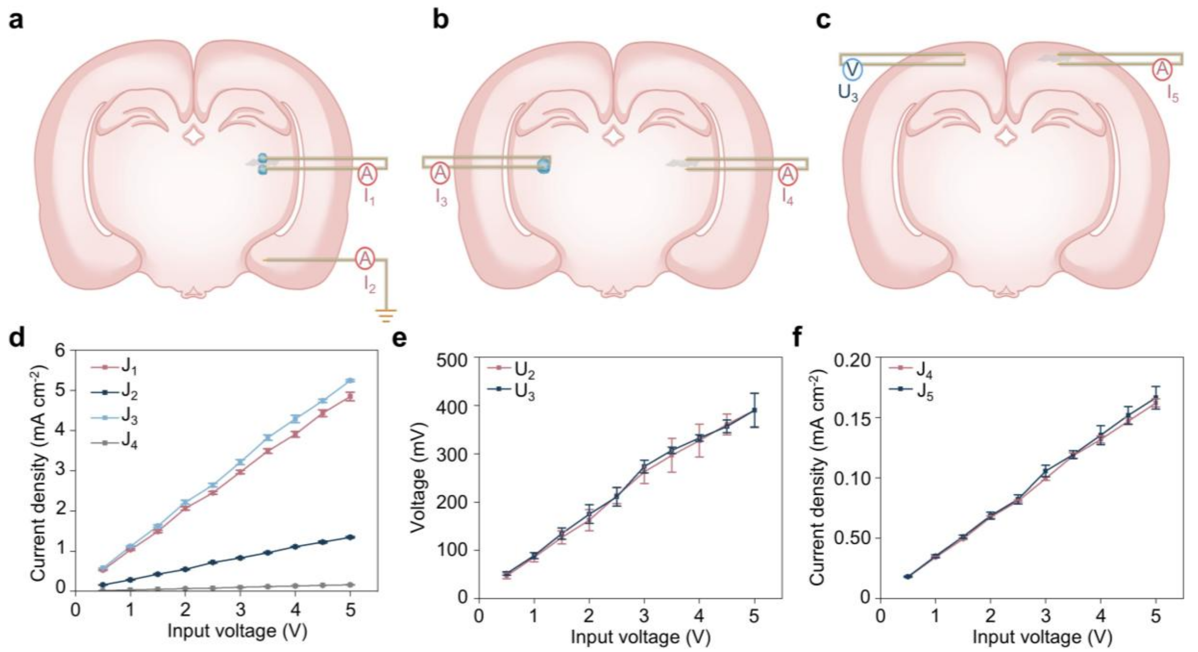

Supplementary Fig. 16. Current output of capacitive coupling system and ICH with varying input voltage. a, Schematic illustration of current measurement points (I_1 and I_2) in the rat brain. I_1 represents the current through the ICHs, I_2 the total current across the entire brain. **b**, Schematic illustration of current measurement points (I_3 and I_4) in the rat brain. I_3 the current measured between the center of the ICHs and the adjacent brain tissue. I_4 serves as a control for I_1, with electrodes placed in brain tissue but separated by an insulating PU membrane, mimicking an interface without ICH-mediated conduction. The electrode depth relative to the external electrode were strictly matched between the I_1 and I_4 conditions, and the only difference between the them was the presence or absence of the ICH. **c**, Schematic of the measurement configuration for cortical voltage (U_3) and current density (J_5), using the same relative electrode spacing as the deep-brain measurements (U_2 and J_4). **d**, Current densities (J_1-J_4) under different input voltages (n = 5 independent experiments). (Note: to account for the contact impedance at the Cu wire-brain tissue interface, the measured values of I_2 and I_4 were multiplied by a correction factor K = 1.5. At 5 MHz, the contact impedance of the hemispherical

electrode was modeled using a parallel RC circuit: The correction factor K, reflecting the ratio of ideal resistive current to actual current, was derived from the impedance magnitude, thus defined as $K = \sqrt{1 + (\omega RC)^2}$). **e**, Voltage measured in cortex (U_3) and deep brain (U_2) under different input voltages (n = 5 independent experiments). **f**, Corresponding current density in cortex (J_5) and deep brain (J_4) under different input voltages (n = 5 independent experiments). Data are presented as the mean ± standard deviation in (**d**, **e**, **f**).

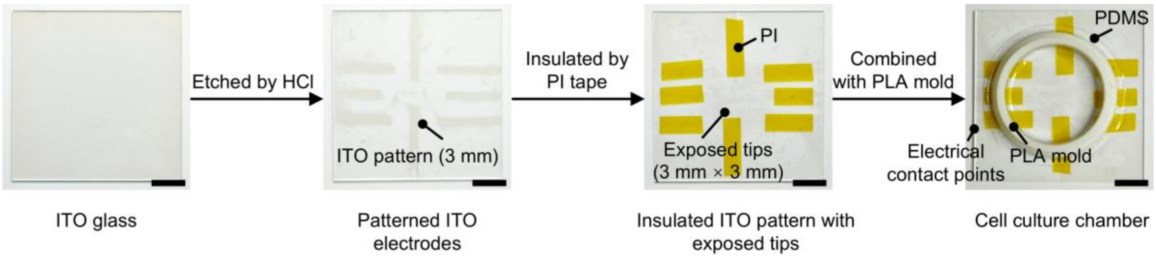

Supplementary Fig. 17. Fabrication of the cell culture chamber with patterned indium tin oxide (ITO) electrodes. The process begins with ITO glass, which is etched using HCl to create patterned ITO electrodes (3 mm wide). The electrodes are then insulated with polyimide (PI) tape, leaving exposed tips (3 mm × 3 mm) for electrical contact. The insulated ITO pattern is subsequently combined with a polylactic acid (PLA) mold and polydimethylsiloxane (PDMS) to form the final cell culture chamber.

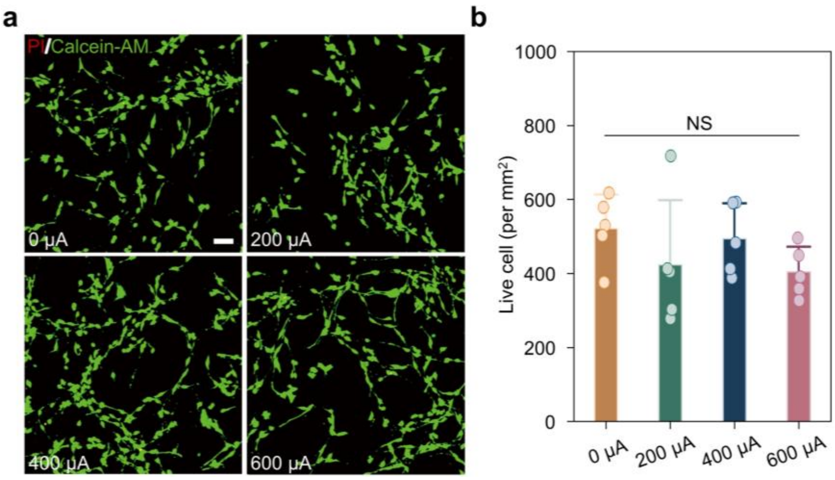

Supplementary Fig. 18. Live-dead staining of PC12 cells cultured on ICH under WES with different current intensities. a, Representative confocal images of live/dead staining of PC12 cells after 3 days of WES at 5 MHz with increasing current amplitudes (0, 200, 400, and 600 μA). Scale bar, 100 μm. **b**, Quantification of live cell density per mm^2 (n = 5 independent experiments), showing no significant decrease in viability up to 600 μA, confirming the biosafety of WES within this range. Data are presented as the mean ± standard deviation in (**b**) and were analyzed by one-way ANOVA first, followed by the Tukey's post hoc test. NS, not significant. **b**, p = 0.6219 (200 μA vs 0 μA), p = 0.7721 (400 μA vs 0 μA), p = 0.9936 (400 μA vs 200 μA), p = 0.9809 (600 μA vs 0 μA), p = 0.4056 (600 μA vs 200 μA), p = 0.5518 (600 μA vs 400 μA).

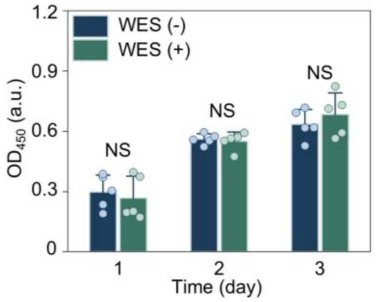

Supplementary Fig. 19. Viability of PC12 cells cultured on ICH with and without WES. PC12 cells were cultured for 3 days with or without WES. The optical density (OD) at 450 nm shows no significant differences between groups (n = 6 independent experiments). Data are presented as mean ± standard deviation and were analyzed using one-way ANOVA, followed by Tukey's post hoc test. NS, not significant. p = 0.6421 (day 1: WES (+) vs WES (-)), p = 0.6700 (day 2: WES (+) vs WES (-)), p = 0.4098 (day 3: WES (+) vs WES (-)).

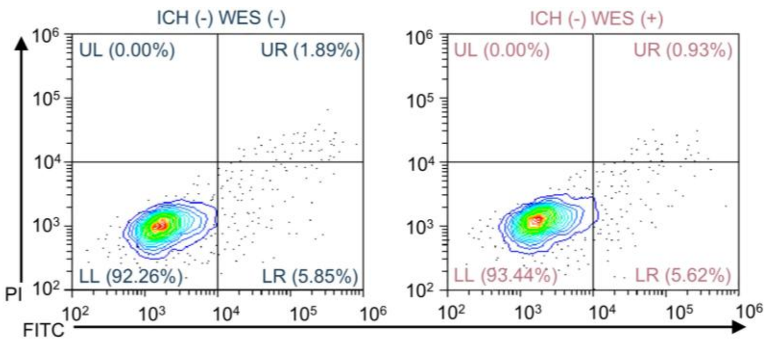

Supplementary Fig. 20. Flow cytometry analysis of PC12 cell viability under ICH (-) conditions. Representative flow cytometry plots of PC12 cells with and without WES treatment.

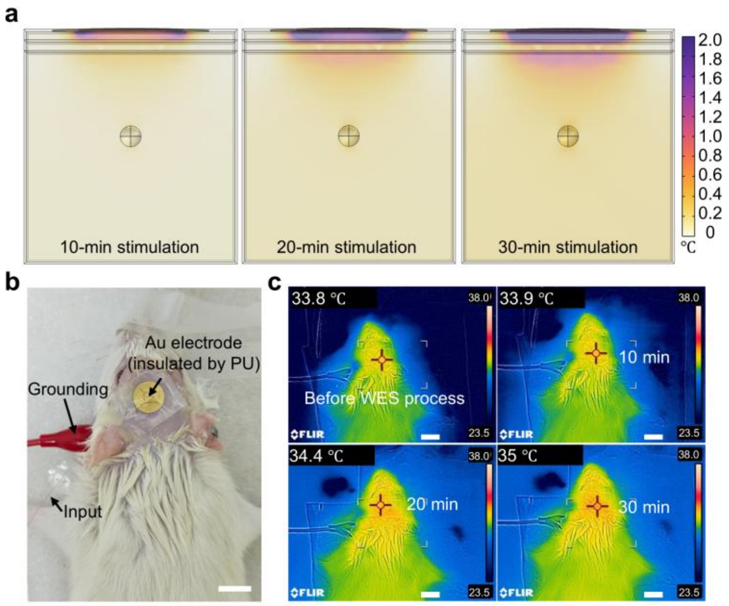

Supplementary Fig. 21. Thermal effects of WES. a, Finite element simulation of temperature distribution in the scalp-skull-brain model after 10-, 20-, and 30-minute WES, showing negligible temperature elevation (< 2 °C). **b**, Photograph of the experimental setup for WES, showing the Au electrode insulated by PU and grounding configuration during stimulation. **c**, Infrared thermographic images of the rat head before and during WES (10 min, 20 min, 30 min), showing minimal change in surface temperature (from 33.8 °C to 35.0 °C). Scale bars, 1 cm.

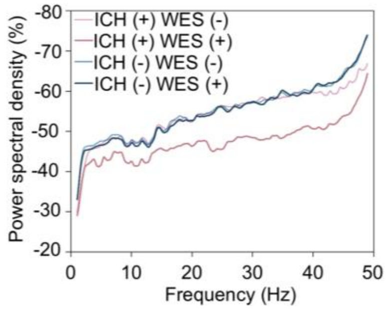

Supplementary Fig. 22. Power spectral density (PSD) of GPi neuronal activity. Neural activity was recorded in four groups: ICH (+) WES (-), ICH (+) WES (+), ICH (-) WES (-), and ICH (-) WES (+). WES stimulation increased PSD primarily in the 10-50 Hz range in the presence of ICH, indicating that the ICH facilitates modulation of GPi oscillatory activity.

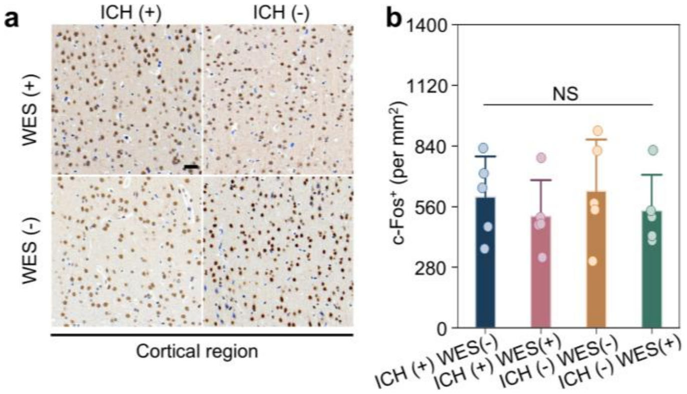

Supplementary Fig. 23. Cellular Fos (c-Fos) expression in the cortical region of rats. a, Representative images of c-Fos expression in the cortical region of rats. Scale bar, 100 μm. The dark brown nuclei represent c-Fos signals, indicating activated neuronal nuclei, while the bluish-purple nuclei correspond to hematoxylin counterstaining, denoting all neuronal nuclei. **b**, Quantification of c-Fos intensity in the cortical region (n = 5 independent animals). Data are presented as the mean ± standard deviation in (**b**) and were analyzed by one-way ANOVA first, followed by the Tukey′s post hoc test. NS, not significant. p_1 (ICH (+) WES (+) vs ICH (+) WES (-)), p_2 (ICH (-) WES (-) vs ICH (+) WES (-)), p_3 (ICH (-) WES (-) vs ICH (+) WES (+)), p_4 (ICH (-) WES (+) vs ICH (+) WES (-)), p_5 (ICH (-) WES (+) vs ICH (+) WES (+)), p_6 (ICH (-) WES (+) vs ICH (-) WES (-)). **b**, $p_1 = 0.8821$, $p_2 = 0.9960$, $p_3 = 0.7757$, $p_4 = 0.9518$, $p_5 = 0.9966$, $p_6 = 0.8767$.

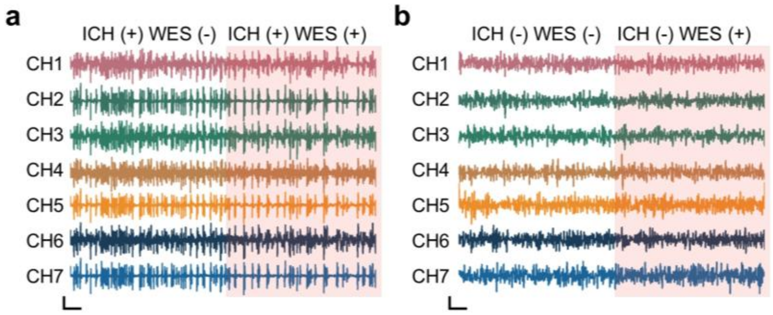

Supplementary Fig. 24. Multi-channel neural recordings from primary motor cortex (M1). a, Neural activity recorded in the presence of ICH before and during WES. **b**, Neural activity recorded without ICH before and during WES. Across channels (CH1-CH7), ICH-assisted WES induced more pronounced modulation in signal amplitude and firing patterns compared to WES alone. Scale bar, 20 s, 100 μV.

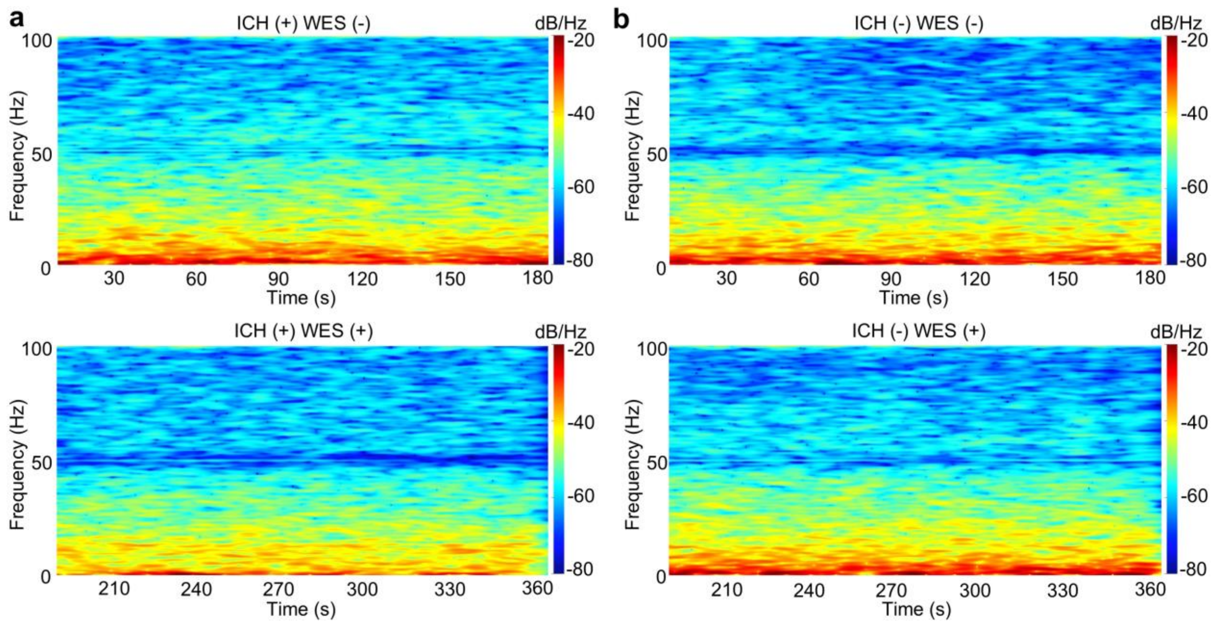

Supplementary Fig. 25. Time-frequency spectrogram of M1 neuronal activity. a, ICH (+) WES (-) induces a more temporally structured oscillatory pattern in the 10-50 Hz range compared to the baseline period (ICH (+) WES (-)). **b**, ICH (-) WES (+) does not markedly alter the spectral pattern relative to baseline (ICH (-) WES (-)).

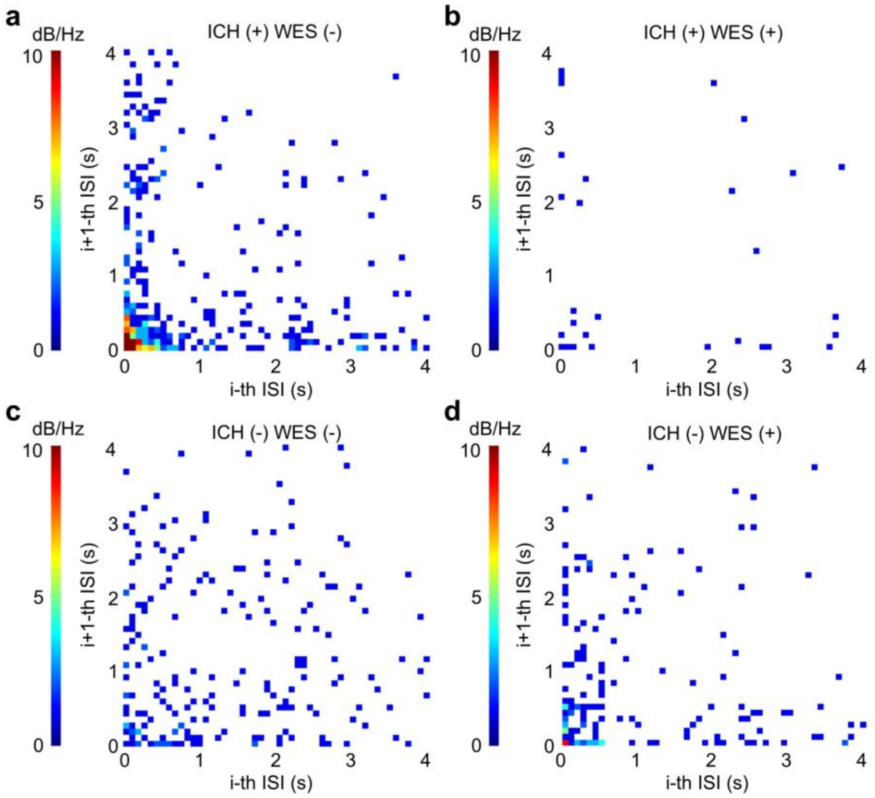

Supplementary Fig. 26. Joint inter-spike interval (ISI) distribution of M1 neuronal firing patterns. a, ICH (+) WES (-): ISI scatter points show a broad distribution, indicating relatively variable spike timing in the baseline state. **b**, ICH (+) WES (+): ISI scatter points become more compact, reflecting increased temporal regularity and reduced spike timing variability following WES when the ICH is present. **c**, ICH (-) WES (-): ISI distribution is widely dispersed, indicating irregular and unstructured neuronal firing without ICH or stimulation. **d**, ICH (-) WES (+): No pronounced change in ISI distribution is observed compared to baseline, suggesting that WES alone does not reorganize spike timing without the ICH.

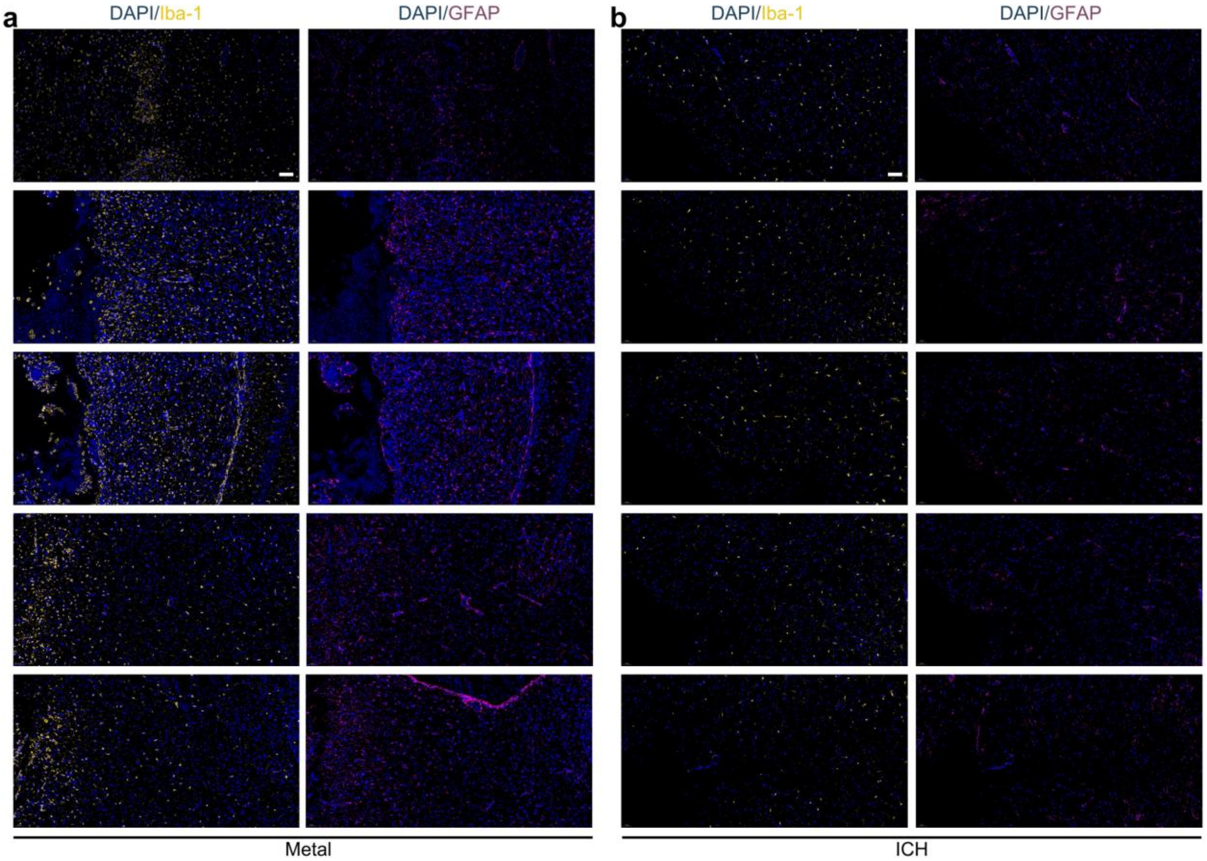

Supplementary Fig. 27. Representative un-cropped confocal images of Iba-1 and GFAP immunostaining used for quantification. **a**, Brain sections surrounding tungsten wire electrodes show dense Iba-1$^+$ microglial activation and GFAP$^+$ astrocytic encapsulation. **b**, Brain sections surrounding ICH show markedly reduced Iba-1 and GFAP fluorescence intensity, indicating minimal inflammatory response. Iba-1, ionized calcium-binding adaptor molecule 1; GFAP, glial fibrillary acidic protein; DAPI, 4',6-diamidino-2-phenylindole. All images were acquired under identical staining and imaging conditions. DAPI, blue. Scale bar, 50 μm.

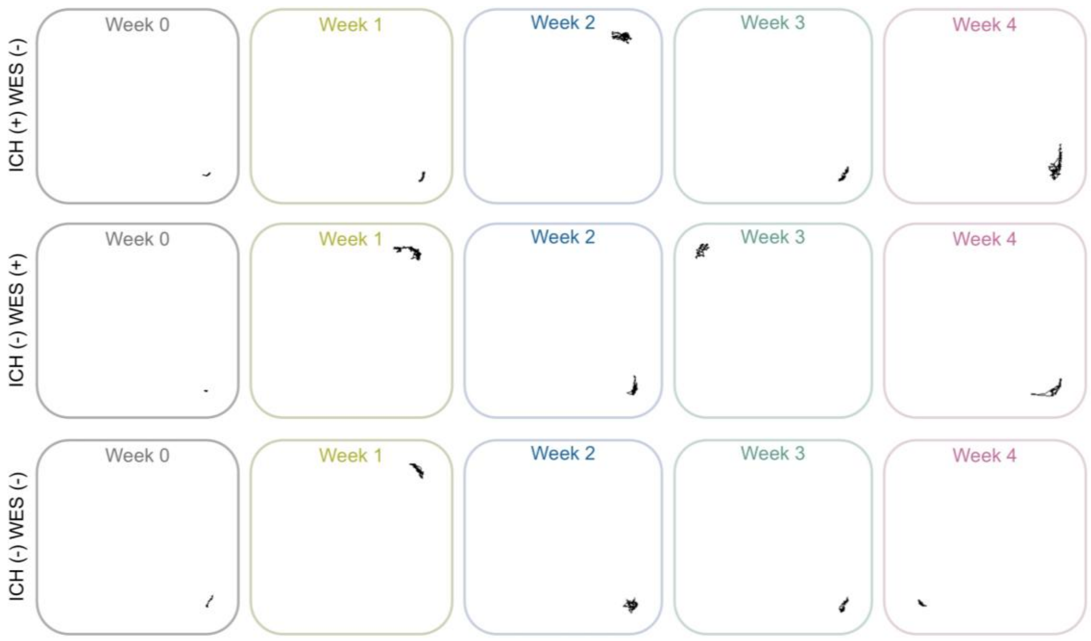

Supplementary Fig. 28. Open field test of PD rats during 4-week treatment. Trajectory images of open field test for evaluation of the locomotor behaviour of PD rats in different groups. The open field test was conducted in a chamber with an automated video tracking system for 5 min.

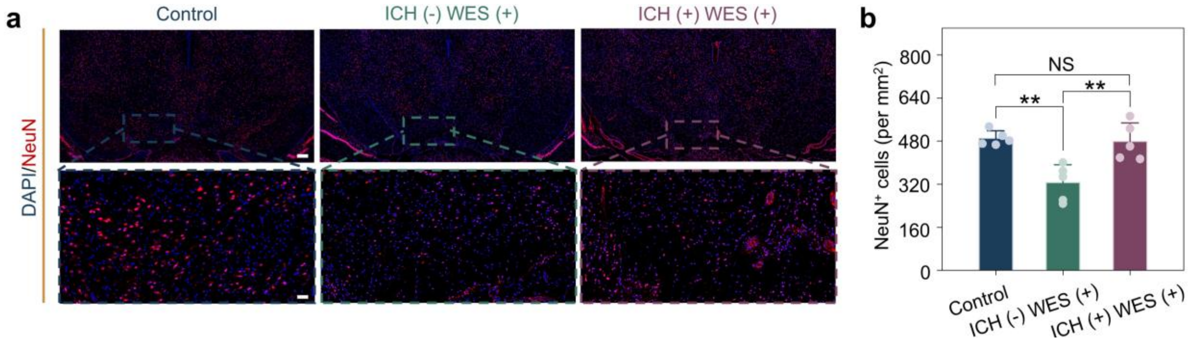

Supplementary Fig. 29. ICH-mediated DBS enhances neuronal survival. a, Representative immunofluorescence images of $NeuN^+$ neurons in the Control, ICHs-BES-free and ICH-BES groups. The bottom images show magnified views of the highlighted regions in top images. Scale bar, 200 μm (top) and 50 μm (bottom). NeuN, neuronal nuclei. **b**, Quantification of $NeuN^+$ cell density (n = 5 independent animals). Data are presented as the mean ± standard deviation in (**b**) and were analyzed by one-way ANOVA first, followed by the Tukey′s post hoc test. $^{**}P \leq 0.01$, NS, not significant. **b**, p = 0.0019 (ICH (-) WES (+) vs Control), p = 0.9432 (ICH (+) WES (+) vs Control), p = 0.0034 (ICH (+) WES (+) vs ICH (-) WES (+)).

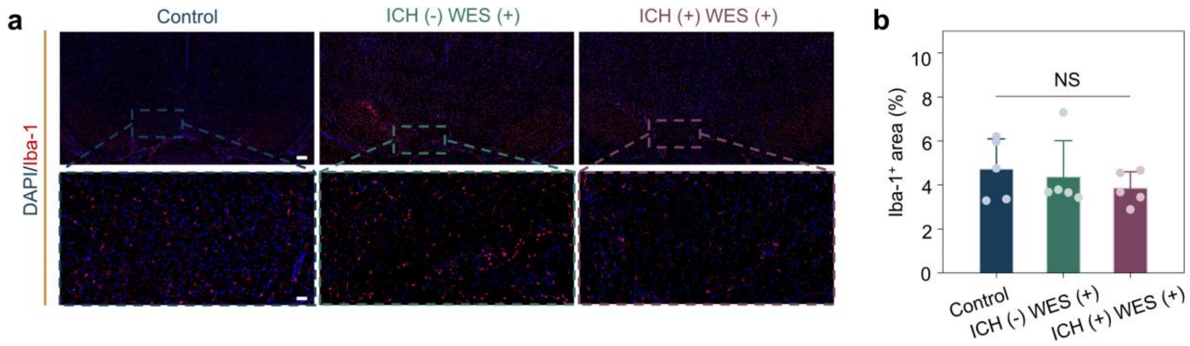

Supplementary Fig. 30. Immunofluorescence analysis of Iba-1^{+} microglia. a, Representative immunofluorescence images of Iba-1^{+} microglia in the Control, ICH-BES-free and ICH-BES groups. The bottom images show magnified views of the highlighted regions in top images. Scale bar, 200 μm (top) and 50 μm (bottom). Iba-1, ionized calcium-binding adaptor molecule 1. **b**, Quantification of Iba-1^{+} area (n = 5 independent animals). Data are presented as the mean ± standard deviation in (**b**) and were analyzed by one-way ANOVA first, and then by the Tukey′s post hoc test. NS, not significant. **b**, p = 0.9097 (ICH (-) WES (+) vs Control), p = 0.5633 (ICH (+) WES (+) vs Control), p = 0.8066 (ICH (+) WES (+) vs ICH (-) WES (+)).

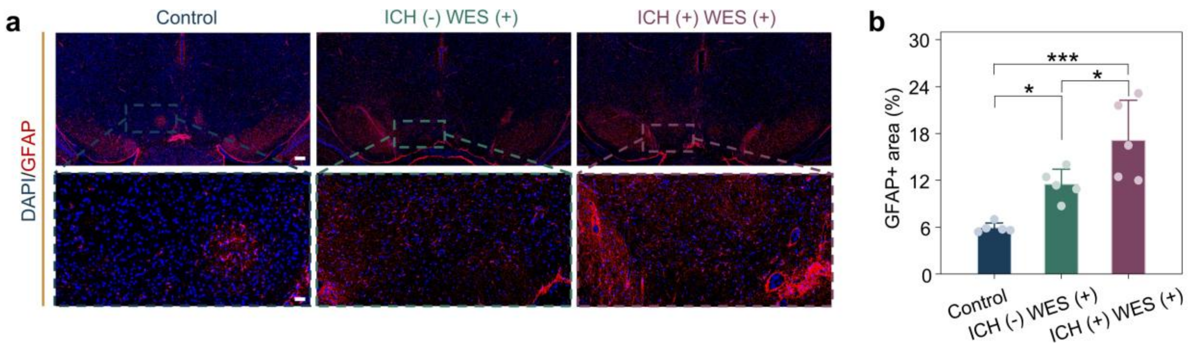

Supplementary Fig. 31. Immunofluorescence analysis of GFAP⁺ astrocytes. a, Representative immunofluorescence images of GFAP⁺ astrocytes in the Control, ICH-BES-free and ICH-BES groups. The bottom images show magnified views of the highlighted regions in top images. Scale bar, 200 μm (top) and 50 μm (bottom). GFAP, glial fibrillary acidic protein. **b**, Quantification of GFAP⁺ area (n = 5 independent animals). Data are presented as the mean ± standard deviation in (**b**) and were analyzed by one-way ANOVA first, and then by the Tukey′s post hoc test. *$P \leq 0.05$, **$P \leq 0.01$, ***$P \leq 0.001$. **b**, p = 0.0424 (ICH (-) WES (+) vs Control), p = 0.0003 (ICH (+) WES (+) vs Control), p = (ICH (+) WES (+) vs ICH (-) WES (+)).

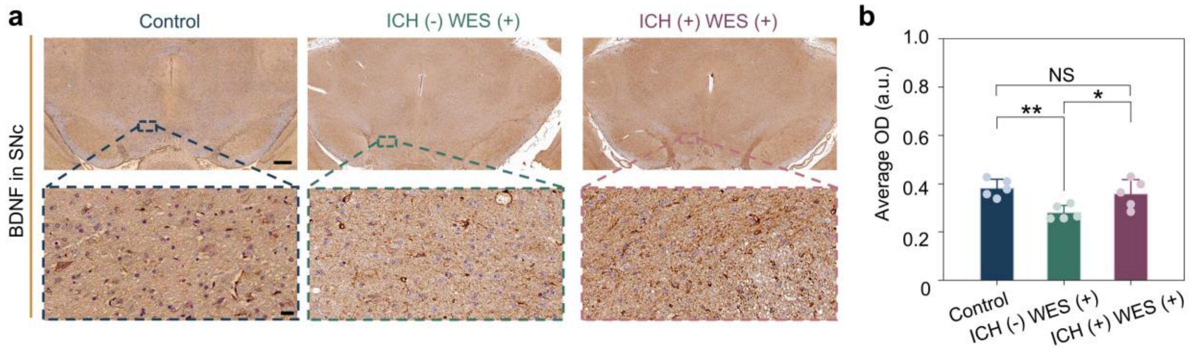

Supplementary Fig. 32. ICH-mediated DBS enhances BDNF expression. a, Representative immunohistochemical images of BDNF in SNc. Scale bar, 500 µm (top) and 20 µm (bottom). BDNF, brain-derived neurotrophic factor. **b**, Average OD analysis of BDNF in SNc (n = 5 independent animals). Data are presented as the mean ± standard deviation in (**b**) and were analyzed by one-way ANOVA first, and then by the Tukey′s post hoc test. *P ≤ 0.05, **P ≤ 0.01, NS, not significant. **b**, p = 0.0083 (ICH (-) WES (+) vs Control), p = 0.677 (ICH (+) WES (+) vs Control), p = 0.0386 (ICH (+) WES (+) vs ICH (-) WES (+)).

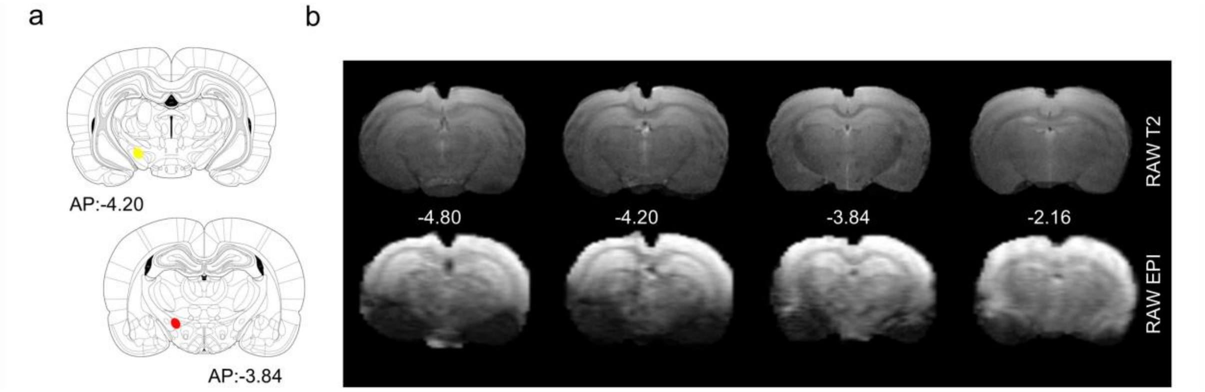

Supplementary Fig. 33. Structural MRI after Parkinson's disease (PD) modeling and injection of ICH. a, Schematic coronal sections illustrating the PD modeling site (yellow dot) and ICH injection site in the subthalamic nucleus (STN; red dot) at anterior-posterior (AP) coordinates of -4.20 mm and -3.84 mm relative to bregma, respectively. **b**, Structural MRI images, including T2-weighted (RAW T2) and echo-planar imaging (RAW EPI), showing the anatomical structure of the brain at AP coordinates of -4.80 mm, -4.20 mm, -3.84 mm, and -2.16 mm.

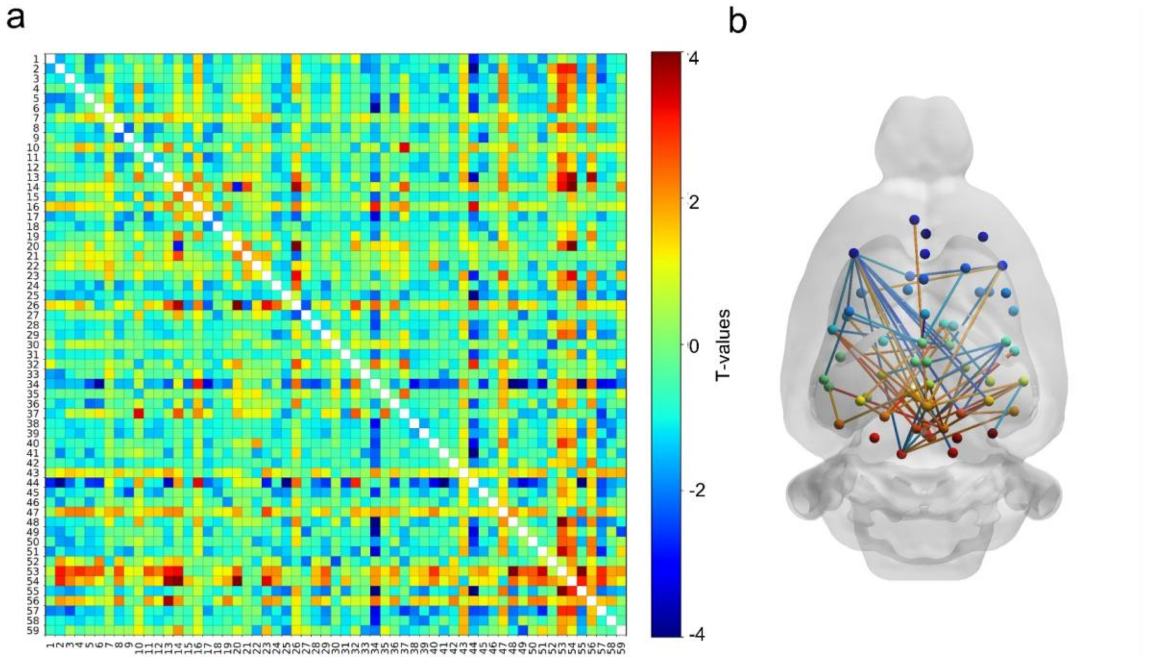

Supplementary Fig. 34. Whole-brain function connectivity analysis. a, Heatmap showing whole-brain functional connectivity across different brain regions. The diagonal represents intra-regional connectivity, while off-diagonal elements indicate inter-regional connectivity patterns. **b**, Three-dimensional functional connectivity networks, with nodes corresponding to brain regions and edges representing significant connections. The color of edges indicates T-values, consistent with the color scale shown in (**a**).

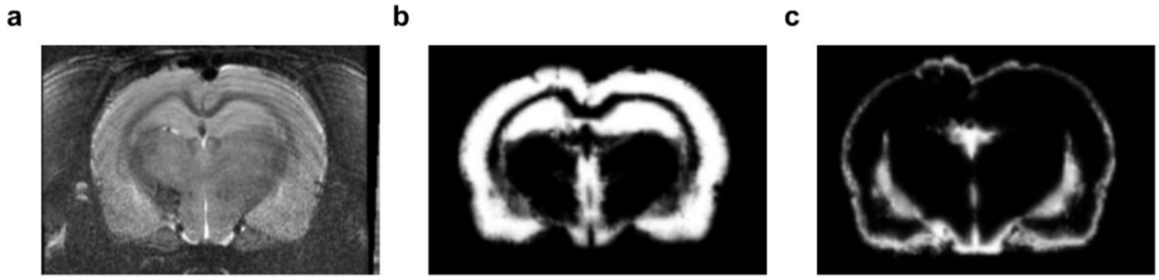

Supplementary Fig. 35. Voxel-based morphometry (VBM) analysis of gray and white matter after ICH-mediated DBS. a, T2-weighted MRI image showing the anatomical structure of the rat brain after 4-week DBS treatment. **b**, VBM analysis of gray matter volume. **c,** VBM analysis of white matter volume.

Supplementary Table 1. Brain region index and anatomical labels.

Index	Brain Region	Index	Brain Region
1	Accumbens Shell (Inter-hemispheric)	31	Retrosplenial Granular Cortex Zone A Postsubiculum (Right)
2	Striatum Dorsal (Left)	32	Retrosplenial Cortex 5 Colliculus (Inter-hemispheric)
3	Striatum Ventral (Left)	33	RSD RSGa (Right)
4	Striatum (Right)	34	Primary Somatosensory (Right)
5	Primary and Secondary Motor (Right)	35	Primary Somatosensory (Left)
6	Amygdala Central Basolateral (Right)	36	Primary Somatosensory Cortex BE (Right)
7	Cingulate Cortex 1 (Inter-hemispheric)	37	Primary Somatosensory Cortex BE (Left)
8	Cingulate Cortex 2 (Inter-hemispheric)	38	Primary Somatosensory Cortex Auditory (Right)
9	Cingulate Cortex 3 (Inter-hemispheric)	39	Parietal Cortex Auditory (Left)
10	Dorsal Hippocampus (Left)	40	Parietal Cortex Auditory (Right)
11	Cornu Ammonis 1 Transition Dorsal Ventral (Left)	41	Secondary Visual Cortex (Inter-hemispheric)
12	Dorsal Hippocampus (Right)	42	Primary and Secondary Visual Cortex (Left)
13	Dorsal Dentate Gyrus (Left)	43	Primary and Secondary Visual Cortex (Right)
14	Cornu Ammonis 1 Ventral (Left)	44	Hypothalamus 1 (Inter-hemispheric)
15	Dorsal Dentate Gyrus Entorhinal Cortex (Right)	45	Hypothalamus 2 (Inter-hemispheric)
16	Insular Cortex (Right)	46	Dorsal Lateral Periaqueductal Gray (Inter-hemispheric)
17	Insular Cortex 2 (Right)	47	Dorsal Thalamic Nucleus (Right)
18	Insular Cortex (Left)	48	Ventral Thalamic (Inter-hemispheric)
19	Prelimbic Cortex (Inter-hemispheric)	49	Medial Geniculate Nucleus (Left)
20	Prelimbic Infralimbic (Inter-hemispheric)	50	Interpeduncular Nucleus (Inter-hemispheric)
21	Endo Piriform Cortex (Left)	51	Inferior Colliculus External Cortex (Right)
22	Piriform Cortex (Right)	52	Ventral Tegmental Nucleus (Inter-hemispheric)
23	Piriform Cortex (Left)	53	Pontine Nuclei (Inter-hemispheric)
24	Intermedial Entorhinal Cortex (Right)	54	Raphe Pallidum Magnus Nuclei (Inter-hemispheric)
25	Retrosplenial Cortex 1 (Inter-hemispheric)	55	Pontine Reticular Subcoeruleus (Left)
26	Retrosplenial Granular Cortex C (Inter-hemispheric)	56	External Colliculus V1 (Left)
27	Retrosplenial Cortex 2 (Inter-hemispheric)	57	Subcoeruleus Pontine Reticular Nucleus (Right)
28	Retrosplenial Cortex 3 Superior Gray (Inter-hemispheric)	58	Colliculus (Right)
29	Retrosplenial Cortex 4 (Inter-hemispheric)	59	Raphe Median Paramedian Pontine Reticular Nucleus (Right)
30	Retrosplenial Granular Cortex Zone A Postsubiculum (Left)		
