# Supplementary material for: Injectable hydrogel bioelectrostimulator for wireless deep brain neuromodulation
Source: Nat Commun. 2026 Feb 4;17:4526. doi: 10.1038/s41467-026-69226-1 (PMC13194742; doi:10.1038/s41467-026-69226-1)
Supplement: Supplementary file 2 — Reporting Summary [file 41467_2026_69226_MOESM2_ESM.pdf]

nature portfolio

Corresponding author(s): Zhiqiang Luo

Last updated by author(s): Jan 1, 2026

Reporting Summary

Nature Portfolio wishes to improve the reproducibility of the work that we publish. This form provides structure for consistency and transparency in reporting. For further information on Nature Portfolio policies, see our Editorial Policies and the Editorial Policy Checklist.

Statistics

For all statistical analyses, confirm that the following items are present in the figure legend, table legend, main text, or Methods section.

n/a	Confirmed	
☐	☒	The exact sample size (*n*) for each experimental group/condition, given as a discrete number and unit of measurement
☐	☒	A statement on whether measurements were taken from distinct samples or whether the same sample was measured repeatedly
☐	☒	The statistical test(s) used AND whether they are one- or two-sided<br>*Only common tests should be described solely by name; describe more complex techniques in the Methods section.*
☒	☐	A description of all covariates tested
☒	☐	A description of any assumptions or corrections, such as tests of normality and adjustment for multiple comparisons
☐	☒	A full description of the statistical parameters including central tendency (e.g. means) or other basic estimates (e.g. regression coefficient) AND variation (e.g. standard deviation) or associated estimates of uncertainty (e.g. confidence intervals)
☐	☒	For null hypothesis testing, the test statistic (e.g. *F*, *t*, *r*) with confidence intervals, effect sizes, degrees of freedom and *P* value noted<br>*Give P values as exact values whenever suitable.*
☒	☐	For Bayesian analysis, information on the choice of priors and Markov chain Monte Carlo settings
☒	☐	For hierarchical and complex designs, identification of the appropriate level for tests and full reporting of outcomes
☐	☒	Estimates of effect sizes (e.g. Cohen's *d*, Pearson's *r*), indicating how they were calculated

Our web collection on statistics for biologists contains articles on many of the points above.

Software and code

Policy information about availability of computer code

Data collection	UV-Vis absorption spectroscopy (SolidSpec-3700, Shimadzu), Nicolet iS50R spectrometer (Thermo Scientific), Laser confocal Raman spectrometer (LabRAM HR800, Horiba Jobin Yvon), Zetasizer Nano ZS90 (Malvern), Rheometer (MCR102, Anton Paar), Four-point probe instrument (KDY-1, Kunde Semiconductor Co., Ltd), Electrochemical workstation (CHI660E, Shanghai Chenhua Apparatus Shanghai Chenhua Co., Ltd), Multichannel potentiostat (CHI1040C, Shanghai Chenhua Apparatus Corporation), Oscilloscope (DHO1204, RIGOL), Power amplifier (ATA-1200B, Aigtek), Function generator (Tektronix, AFG3021C), Current amplifier (OE4102, Sine Scientific Instruments), Confocal microscopy (FV3000, Olympus), Multi-function microplate reader (PARK 10M, TECAN), Flow cytometer (CytoFLEX, Beckman Coulter), RHS Stim/Recording System (RHS2000, Intan Technologies), Infrared thermographic imaging system (E5 Pro, FLIR), SMART video tracking system (SMART 3.0, Panlab), 7T MRI scanner (BioSpin, Bruker)
Data analysis	Origin 2023 software was used for data plotting and statistical analysis. Image J software (v1.54m) was used for quantitative imaging analyses. COMSOL Multiphysics® software (v6.3) was used for finite element simulation. The collected electrophysiological data were analyzed using NeuroExplorer (Nex Technologies, v5) and Offline Sorter (Plexon, v4.7.3). fMRI data preprocessing and analysis were performed using AFNI, FSL, and ANTs software packages (v2.4.4), supplemented by MATLAB scripts (R2019b). ITK-SNAP (v4.0.2) was employed for semi-manual skull stripping, and BrainNet Viewer was used for network visualization.

For manuscripts utilizing custom algorithms or software that are central to the research but not yet described in published literature, software must be made available to editors and reviewers. We strongly encourage code deposition in a community repository (e.g. GitHub). See the Nature Portfolio guidelines for submitting code & software for further information.

Data

Policy information about availability of data

All manuscripts must include a data availability statement. This statement should provide the following information, where applicable:

- Accession codes, unique identifiers, or web links for publicly available datasets
- A description of any restrictions on data availability
- For clinical datasets or third party data, please ensure that the statement adheres to our policy

All data supporting the findings of this study are available within the article and its supplementary files. Any additional requests for information can be directed to, and will be fulfilled by, the corresponding authors. Source data are provided with this paper.

Research involving human participants, their data, or biological material

Policy information about studies with human participants or human data. See also policy information about sex, gender (identity/presentation), and sexual orientation and race, ethnicity and racism.

Reporting on sex and gender	This work did not involve human research.
Reporting on race, ethnicity, or other socially relevant groupings	This work did not involve human research.
Population characteristics	This work did not involve human research.
Recruitment	This work did not involve human research.
Ethics oversight	This work did not involve human research.

Note that full information on the approval of the study protocol must also be provided in the manuscript.

Field-specific reporting

Please select the one below that is the best fit for your research. If you are not sure, read the appropriate sections before making your selection.

- [x] Life sciences
- [ ] Behavioural & social sciences
- [ ] Ecological, evolutionary & environmental sciences

For a reference copy of the document with all sections, see nature.com/documents/nr-reporting-summary-flat.pdf

Life sciences study design

All studies must disclose on these points even when the disclosure is negative.

Sample size	Details regarding sample sizes for all experiments are provided in the Methods section and figure legends. Sample sizes were chosen based on prior experience with similar experimental systems, consistency with related published studies, and practical considerations typical for exploratory bioelectronics and in vivo experiments. For material characterization and in vitro experiments, each condition was tested using at least three independent samples or experiments, which was sufficient to ensure reproducibility and allow statistical comparison between groups. For in vivo animal experiments, a sample size of five rats per group was used, which is consistent with previous studies in the field and sufficient to detect robust stimulation-induced and behavioral effects at a significance level of 0.05. For MRI experiments, nine rats were initially included; one animal was excluded due to fixation-related artifacts that compromised image quality, resulting in a final sample size of eight animals for MRI analysis.
Data exclusions	No data were excluded from this study.
Replication	All experiments were performed with independent replicates as described in the figure legends.
Randomization	All samples were randomly allocated into experimental groups.
Blinding	Investigators were blinded to group allocation during data collection and analysis.

Reporting for specific materials, systems and methods

We require information from authors about some types of materials, experimental systems and methods used in many studies. Here, indicate whether each material, system or method listed is relevant to your study. If you are not sure if a list item applies to your research, read the appropriate section before selecting a response.

Materials & experimental systems

n/a	Involved in the study	
☐	☒	Antibodies
☐	☒	Eukaryotic cell lines
☒	☐	Palaeontology and archaeology
☐	☒	Animals and other organisms
☒	☐	Clinical data
☒	☐	Dual use research of concern
☒	☐	Plants

Methods

n/a	Involved in the study	
☒	☐	ChIP-seq
☐	☒	Flow cytometry
☐	☒	MRI-based neuroimaging

Antibodies

Antibodies used

Primary antibodies:
1) Anti-Iba1 Rabbit pAb (Iba-1; Recombinant protein corresponding to Mouse Iba1; Servicebio; Catalog No. GB113502; dilution 1:500); Species Reactivity: Human, Mouse, Rat, Application: IHC/IF;
2) Anti-GFAP Rabbit mAb (GFAP; Recombinant protein corresponding to Mouse GFAP; Servicebio; Catalog No. GB15100; dilution 1:1000); Species Reactivity: Human, Mouse, Rat, Application: WB, IHC/IF;
3) Anti-c-Fos Mouse mAb (c-Fos; Recombinant protein corresponding to Mouse c-Fos; Bosterbio; Catalog No. M00297-6; dilution 1:250); Species Reactivity: Human, Mouse, Rat, Application: IHC;
4) Anti-NeuN Rabbit pAb (NeuN; Recombinant protein corresponding to Mouse NeuN; Servicebio; Catalog No. GB11138; dilution 1:300); Species Reactivity: Human, Mouse, Rat, Application: WB, IHC/IF;
5) Anti-Tyrosine Hydroxylase Rabbit pAb (TH; Recombinant protein corresponding to Mouse Tyrosine Hydroxylase; Catalog No. GB11181; dilution 1:1000); Species Reactivity: Human, Mouse, Rat, Application: WB, IHC/IF;
6) Anti-BDNF Rabbit pAb (BDNF; Recombinant protein corresponding to Mouse BDNF; Catalog No. GB11559; dilution 1:100); Species Reactivity: Human, Mouse, Rat, Application: WB, IHC/IF;

Secondary antibodies:
1) HRP conjugated Goat Anti-Mouse IgG (H+L) (Servicebio; Catalog No. GB23301; dilution 1:200); Species Reactivity: Mouse, Rat, Application: Elisa, WB, IHC;
2) Cy3 conjugated Goat Anti-Rabbit IgG (H+L) (Excitation and emission wavelength: 550nm and 570nm; Servicebio; Catalog No. GB21303; dilution 1:300); Species Reactivity: Mouse, Rat, Application: IF/FC;
3) Cy3 conjugated Goat Anti-Mouse IgG (H+L) (Excitation and emission wavelength: 550nm and 570nm; Servicebio; Catalog No. GB21301; dilution 1:300); Species Reactivity: Mouse, Rat, Application: IF/FC;
4) Alexa Fluor 488-conjugated Goat Anti-Rabbit IgG (H+L) (Excitation and emission wavelength: 493nm and 519nm; Servicebio; Catalog No. GB25303; dilution 1:400); Application: IF/FC

Validation

All primary antibodies used in this study were commercially sourced and validated by the manufacturers for immunohistochemistry and/or immunofluorescence. Antibody validation information, including species reactivity, recommended dilutions, and representative application data, is provided on the manufacturers' official websites.

Eukaryotic cell lines

Policy information about cell lines and Sex and Gender in Research

Cell line source(s)

PC12 cell lines (catalog number: QS-R009) and SH-SY5Y cell lines (catalog number: QS-H036) were purchased from Keycell Biotechnology (Hubei, China).

Authentication

PC12 and SH-SY5Y cell lines were not authenticated by the investigators in this paper. They were purchased from the company.

Mycoplasma contamination

PC12 and SH-SY5Ycell lines were not tested for mycoplasma contamination.

Commonly misidentified lines
(See ICLAC register)

No commonly misidentified cell lines were used in the study.

Animals and other research organisms

Policy information about studies involving animals; ARRIVE guidelines recommended for reporting animal research, and Sex and Gender in Research

Laboratory animals

Male Sprague Dawley rats (SD rats, ~200 g, 7-week-old) were used for all our experiments.

Wild animals

This study did not involve wild animals.

Reporting on sex

Only male rats were used to minimize variability associated with hormonal cycles and estrogen-related neuroprotection, which can influence dopaminergic signaling and behavioral outcomes in females

Field-collected samples	This study did not involve samples collected from the field.
Ethics oversight	All animal experiments were conducted in accordance with guidelines approved by the Institutional Animal Care and Use Committee of Huazhong University of Science and Technology (TJH-202212040).

Note that full information on the approval of the study protocol must also be provided in the manuscript.

Plants

Seed stocks	This study did not involve seed stocks.
Novel plant genotypes	This study did not involve plant genotypes.
Authentication	This study did not involve seed stocks or plant genotypes.

Flow Cytometry

Plots

Confirm that:

- [x] The axis labels state the marker and fluorochrome used (e.g. CD4-FITC).
- [x] The axis scales are clearly visible. Include numbers along axes only for bottom left plot of group (a 'group' is an analysis of identical markers).
- [x] All plots are contour plots with outliers or pseudocolor plots.
- [x] A numerical value for number of cells or percentage (with statistics) is provided.

Methodology

Sample preparation	PC12 cells were harvested after stimulation, washed twice with phosphate-buffered saline (PBS), and stained with Annexin V-FITC and propidium iodide (PI) according to the manufacturer's protocol to evaluate apoptosis and necrosis.
Instrument	Flow cytometry analysis was performed using a CytoFLEX flow cytometer (Beckman Coulter, USA).
Software	Data acquisition and analysis were conducted using CytExpert software (Beckman Coulter). Percentages of viable (Annexin V^-/PI^-), early apoptotic (Annexin V^+/PI^-), and late apoptotic/necrotic (Annexin V^+/PI^+) populations were quantified.
Cell population abundance	Apoptotic and necrotic cell populations were quantified as a percentage of total cell events (>10,000 events per sample). Data are presented as mean ± SD from three independent experiments.
Gating strategy	Gating was based on forward scatter (FSC) and side scatter (SSC) to exclude debris and aggregates. Quadrant gating on Annexin V-FITC and PI channels was used to distinguish viable, early apoptotic, and late apoptotic/necrotic populations. Gates were defined using single-stained and unstained controls for compensation and boundary determination.

- [ ] Tick this box to confirm that a figure exemplifying the gating strategy is provided in the Supplementary Information.

Magnetic resonance imaging

Experimental design

Design type	Resting-state fMRI with an event-related design.
Design specifications	Each imaging session consisted of a 20 min single-run fMRI acquisition with 600 EPI volumes (TR = 2 s). A total of 9 rats were scanned, with one excluded due to fixation-related motion artifacts.
Behavioral performance measures	No behavioral task was performed. All animals were anesthetized to minimize motion and physiological variability. Physiological parameters (respiratory rate, heart rate, and temperature) were continuously monitored to ensure stable resting-state conditions throughout the experiment.

Acquisition

Imaging type(s)	Functional and structural MRI.
Field strength	7 Tesla
Sequence & imaging parameters	Structural images were acquired using a TurboRARE sequence (FOV = 20.35 × 21.00 mm², matrix = 256 × 256, RARE factor = 8, TR = 6800 ms, TE = 12 ms, effective TE = 36 ms, averages = 8, flip angle = 180°, slice thickness = 0.5 mm). Functional images were obtained using single-shot echo planar imaging (EPI) (TR = 2000 ms, TE = 15.32 ms, segments = 1, matrix = 64 × 64, in-plane resolution = 125 × 125 µm², slice thickness = 500 µm, 32 slices, 600 volumes over 20 min).
Area of acquisition	Whole-brain acquisition covering the entire rat cerebrum and brainstem.

Diffusion MRI ☐ Used ☒ Not used

Preprocessing

Preprocessing software	AFNI (NIH, USA), FSL (Analysis Group, FMRIB, Oxford, UK), and ANTs (Advanced Normalization Tools; https://github.com/ANTsX/ANTs) were used, supplemented by MATLAB (MathWorks, USA) scripts. Skull stripping was performed semi-manually using ITK-SNAP, brain masking with FSL's fslmaths, registration with ANTs (antsRegistrationSyN.sh), bias correction with N4BiasFieldCorrection, denoising with DenoiseImage, motion correction with antsMotionCorr, and temporal shift correction with AFNI's 3dTshift. Spatial smoothing was performed with a Gaussian kernel (0.4 mm isotropic, twice the voxel size).
Normalization	Both linear and nonlinear spatial normalization were applied to align individual EPI images to a study-specific mouse template and subsequently to the standard TMBTA rat brain template.
Normalization template	TMBTA rat brain template (https://www.nitrc.org/projects/tmbta) and study-specific intermediate template based on the Allen Brain Atlas (https://atlas.brain-map.org/).
Noise and artifact removal	Physiological noise (e.g., respiration and cardiac cycle) and scanner drifts were reduced using motion correction and temporal filtering steps integrated in AFNI and ANTs. Visual inspection was performed to confirm the absence of ghosting or motion artifacts after preprocessing.
Volume censoring	Volumes exhibiting motion greater than 0.2 mm displacement or abrupt signal intensity spikes were excluded from analysis (<5% of total volumes per animal).

Statistical modeling & inference

Model type and settings	Functional connectivity analysis was performed using Pearson's correlation to construct 59 × 59 connectivity matrices among predefined regions of interest (ROIs). For each ROI, mean time series were extracted using AFNI's 3dNetCorr tool. Group-level comparisons between stimulation and control conditions were assessed using two-sample t-tests implemented in MATLAB, following Fisher's z-transformation of correlation coefficients.
Effect(s) tested	Differences in functional connectivity strength before and after treatment.

Specify type of analysis: ☐ Whole brain ☒ ROI-based ☐ Both

Anatomical location(s)	Anatomical regions of interest (ROIs) were defined according to the SIGMA Rat Brain Template and Atlas (https://www.nitrc.org/projects/sigma_template, Version 1-2-1). Each ROI was manually aligned to the TMBTA standard brain template after EPI registration to ensure anatomical consistency. Automated labeling of brain regions was performed using the atlas-based parcellation masks, and ROI boundaries were verified through visual inspection in FSLeyes.
Statistic type for inference (See Eklund et al. 2016)	Two-sample t-test with family-wise error correction (FWE) applied to each ROI–ROI connection.
Correction	Family-wise error (FWE) correction ($p < 0.05$) was used to control for multiple comparisons across the 59 × 59 connectivity matrix. Significant ROI pairs were visualized using chord diagrams and BrainNet Viewer.

Models & analysis

n/a	Involved in the study
☐	☒ Functional and/or effective connectivity
☒	☐ Graph analysis
☒	☐ Multivariate modeling or predictive analysis

Functional and/or effective connectivity	Functional connectivity was assessed using Pearson's correlation between the mean fMRI time series of each pair of predefined ROIs. Correlation coefficients were Fisher's z-transformed prior to group-level statistical analysis. Connectivity matrices (59 × 59) were constructed for each subject to evaluate pairwise functional coupling between brain regions ipsilateral to the stimulation site.
